# Dynamic light scattering for particle characterization subjected to ultrasound: a study on compact particles and acousto-responsive microgels

**DOI:** 10.1038/s41598-024-51404-0

**Published:** 2024-01-10

**Authors:** Sebastian Stock, Regine von Klitzing, Amin Rahimzadeh

**Affiliations:** Soft Matter at Interfaces, Institute for Condensed Matter Physics, Technische Universität Darmstadt, Hochschulstraße 8, 64289 Darmstadt, Germany

**Keywords:** Phase transitions and critical phenomena, Techniques and instrumentation, Acoustics

## Abstract

In this report, we investigate dynamic light scattering (DLS) from both randomly diffusing silica particles and acousto-responsive microgels in aqueous dispersions under ultrasonic vibration. Employing high-frequency ultrasound (US) with low amplitude ensures that the polymers remain intact without damage. We derive theoretical expressions for the homodyne autocorrelation function, incorporating the US term alongside the diffusion term. Subsequently, we successfully combined US with a conventional DLS system to experimentally characterize compact silica particles and microgels under the influence of US. Our model allows us to extract essential parameters, including particle size, frequency, and amplitude of particle vibration, based on the correlation function of the scattered light intensity. The studies involving non-responsive silica particles demonstrate that the US does not disrupt size determination, establishing them as suitable reference systems. In addition, we could be able to experimentally resolve the µs-order motion of particles for the first time. Microgels subjected to the US show the same swelling/shrinking behavior as that induced by temperature but with significantly faster kinetics. The findings of this study have potential applications in various industrial and biomedical fields such as smart coatings and drug delivery that benefit from the characterization of macromolecules subjected to the US. Furthermore, the current work may lead to characterizing the mechanical properties of soft particles based on their vibration amplitude extracted using this method.

## Introduction

Dynamic light scattering (DLS) is commonly used for the characterization of particles and molecules in solutions/dispersions, such as determining their size, size distribution as well as conformational changes. DLS offers to measure sub-micrometer (from a few nanometers to one micrometer) particles accurately. When a laser beam impinges the particles inside a liquid sample, the particles scatter light in all directions. A detector at a certain location detects a fraction of the scattered light and measures it as intensity fluctuations over time. In a conventional DLS system, these fluctuations are caused by interference between scattered light from an ensemble of particles moving due to Brownian motion. The intensity fluctuations can be analyzed by calculating the time-dependent correlation of the signal with itself in different lag times which is called the autocorrelation analysis technique. The particle size can be determined by the decay rate of the autocorrelation function according to the Stokes–Einstein relation^1,2^. According to the Brownian motion theory, smaller particles diffuse faster and thus exhibit shorter correlation times^3^.

In many cases, information about the mechanical, conformational and electrical properties of particles or molecules, including their size, shape, and surface charges are needed under the influence of external forces or fields. Therefore, *dynamic light scattering in external fields* has many applications in biophysics, materials science, and chemical engineering^4^. In those systems– e.g., particles in alternating electric fields^5,6^, in thermal in-homogeneities^7,8^, or directional flows^9^– colloidal particles undergo another movement in addition to their original random motion. These additional movements create complicated intensity fluctuations from which the autocorrelation function (ACF)– with the conventional fitting parameters– does not impart the diffusion coefficient and subsequently the correct particle size anymore^10^. Therefore, one should modify the ACF fitting parameter in order to include the additional translational motion and to distinguish it from the pure Brownian diffusion. For instance, in the case of DLS measurements of colloidal particles in a flowing condition, researchers have modified the ACF so that they could obtain the correct particle size as well as the flowing velocity^9,11–13^. They incorporated a term to account for translational motion under the assumption that it significantly exceeds the diffusion length. One of the external fields that have drawn attention in recent decades is the US which is employed to manipulate the physical or chemical properties of particles and molecules with applications in drug delivery^14^, catalysis^15^ and materials synthesis^16^. Recently, we showed that the high-frequency US, in its non-destructive condition (low amplitude), can be used as a stimulus to induce a phase transition in solutions of linear poly(N-Isopropylacrylamide)(PNIPAM)^17,18^ and also PNIPAM microgels at an oil-water interface^19^. The dehydration of PNIPAM which is usually triggered by increasing the temperature above the lower critical solution temperature (LCST), is then induced by the US. In the case of linear PNIPAM, the phase transition is detectable by an onset of turbidity. PNIPAM microgels are cross-linked polymer networks that upon a stimulus shrink and reduce in size having a promising application in drug delivery systems. They are also temperature sensitive and their dehydration might be also induced by the US. In order to monitor the microgel size (or any other responsive particles) subjected to the US, one has to modify the DLS system and analyze the resulting ACF of standard particles under the influence of ultrasonic waves as a reference system. Besides volume phase transition (VPT), ultrasound might lead to other disturbances like fluctuation of the microgels' trajectory or acoustic streaming, which might induce an apparent size change of the microgels. In order to separate these effects, in this work, first we determine the size of compact particles using DLS subjected to US, assuming that they are shape invariant in US. In the first step, we derive the homodyne correlation function for vibrating particles based on the continuity equation in the work done by Berne and Pecora^20^. The resulting model excludes the assumption in works of references^9,11–13^ and includes information about the particles' vibration frequency and amplitude which can help to resolve μs-ranged particle motion. Finally, we compare the results to a representative system of silica nanospheres subjected to US. This system is a simple combination of a conventional DLS setup with a US component that can establish a ground for more complicated experimental systems.

Based on the evaluated results of the reference system, we conduct an experiment using a sample of PNIPAM microgels to assess their US-induced VPT using their changes in size. The change in microgel diameter during ultrasonic actuation is compared to its diameter variation due to temperature changes. The potential acousto-responsiveness of PNIPAM microgels can find exciting applications in the realm of smart coatings. By assembling 2D crystals of the stimuli-responsive microgels bounded on solid surfaces, functional materials with versatile uses, including sensors, active optical devices, and more can be developed^21–23^. The US as a fast stimulus with tunable spatiotemporal imposition holds significant potential for further advancements in this field.

## Deriving equations

Here we show the principal derivation of the intensity autocorrelation function of the scattered light from an ensemble of particles undergoing Brownian diffusional motion as well as an external-induced flow motion with the velocity ${\varvec{v}}$*.* The particle concentration at point ${\varvec{r}}$ and time $t$ is defined by $c({\varvec{r}},t)$. The continuity equation which describes how the particles flow and diffuse (having a diffusion coefficient ${\text{D}}$) in the system can be written as:

1 $$\frac{\partial c}{\partial t}+\nabla \cdot \left({\varvec{v}}c\right)=D{\nabla }^{2}c$$

According to Berne and Pecora^20^, whose notation we adopted in this study, it is reasonable to assume that the *probability distribution function*, ${G}_{s}({\varvec{r}},t)$, satisfies the same equation. Therefore, we have:

2 $$\frac{\partial {G}_{s}}{\partial t}+\nabla \cdot \left({{\varvec{v}}G}_{s}\right)=D{\nabla }^{2}{G}_{s}$$

We consider the *characteristic function of distribution*, ${F}_{s}({\varvec{q}},t)$ as the Fourier transform of ${G}_{s}$, based on the following definitions:

3 $${F}_{s}\left({\varvec{q}},t\right)=\int {G}_{s}\left({\varvec{r}},t\right){\text{exp}}\left(i{\varvec{q}}\cdot {\varvec{r}}\right){d}^{3}r,$$

4 $${G}_{s}\left({\varvec{r}},t\right)={\left(2\pi \right)}^{-3}\int {F}_{s}\left({\varvec{q}},t\right){\text{exp}}\left(-i{\varvec{q}}\cdot {\varvec{r}}\right){d}^{3}q$$

where $q=\frac{4\pi n\mathrm{ sin}\left(\frac{\theta }{2}\right)}{{\lambda }_{l}}$ is the scattering wave vector with a wavelength of ${\lambda }_{l}$ and scattering angle of $\theta$ and the medium refractive index of *n*. In case the system is subjected to US waves having a wave vector of $\left|{\varvec{k}}\right|=2\pi /{\lambda }_{u}$ (${\lambda }_{u}$ is the US wavelength) and angular frequency of $\omega$, the velocity of the fluid at point ${\varvec{r}}$ and time $t$, having a mean value ${{\varvec{v}}}_{0}$ can be written as:

5 $${\varvec{v}}\left({\varvec{r}},t\right)={{\varvec{v}}}_{0}{\text{exp}}\left(i{\varvec{k}}\cdot {\varvec{r}}-i\omega t\right).$$

By taking the spatial Fourier transform from Eq. (2), the first term on the left-hand side and the term on the right-hand side yield to $\frac{\partial {F}_{s}({\varvec{q}},t)}{\partial t}$ and $-D{q}^{2}{F}_{s}\left({\varvec{q}},t\right)$, respectively. The second term on the left-hand side can be written as (Ft: Fourier transform):

6 $$Ft\left[{\varvec{v}}\cdot \nabla G+G\nabla \cdot {\varvec{v}}\right]=\int {\varvec{v}}\cdot \frac{\partial {G}_{s}}{\partial r}{\text{exp}}\left(i{\varvec{q}}\cdot {\varvec{r}}\right){d}^{3}r+\int {G}_{s}\frac{\partial {\varvec{v}}}{\partial r}{\text{exp}}\left(i{\varvec{q}}\cdot {\varvec{r}}\right){d}^{3}r.$$

Using the Eq. (5), the Eq. (6) yields to:

7 $${{\varvec{v}}}_{0}{\text{exp}}\left(-i\omega t\right)\int \frac{\partial {G}_{s}}{\partial r}{{\text{e}}}^{i\left({\varvec{q}}+{\varvec{k}}\right)\cdot {\varvec{r}}}{d}^{3}r+i{\varvec{k}}{\cdot {\varvec{v}}}_{0}{\text{exp}}\left(-i\omega t\right)\int {G}_{s}{{\text{e}}}^{i\left({\varvec{q}}+{\varvec{k}}\right)\cdot {\varvec{r}}}{d}^{3}r,$$

where $\int \frac{\partial {G}_{s}}{\partial r}{{\text{e}}}^{i\left({\varvec{q}}+{\varvec{k}}\right)\cdot {\varvec{r}}}{d}^{3}r=-iq\int {G}_{s}{{\text{e}}}^{i\left({\varvec{q}}+{\varvec{k}}\right)\cdot {\varvec{r}}}{d}^{3}r$. The reason is that taking a derivative from Eq. (4), leads to:

8 $$\frac{\partial }{\partial r}{G}_{s}\left({\varvec{r}},t\right)={-iq\left(2\pi \right)}^{-3}\int {F}_{s}\left({\varvec{q}},t\right){\text{exp}}\left(-i{\varvec{q}}\cdot {\varvec{r}}\right){d}^{3}q=-iq{G}_{s}.$$

Therefore, Eq. (7) can be written as:

9 $$-i{\varvec{q}}\cdot {{\varvec{v}}}_{0}{\text{exp}}\left(-i\omega t\right)\int {G}_{s}{{\text{e}}}^{i\left({\varvec{q}}+{\varvec{k}}\right)\cdot {\varvec{r}}}{d}^{3}r+i{\varvec{k}}\cdot {{\varvec{v}}}_{0}{\text{exp}}\left(-i\omega t\right)\int {G}_{s}{{\text{e}}}^{i\left({\varvec{q}}+{\varvec{k}}\right)\cdot {\varvec{r}}}{d}^{3}r=-i\left({\varvec{q}}-{\varvec{k}}\right)\cdot {{\varvec{v}}}_{0}{\text{exp}}\left(-i\omega t\right){F}_{s}\left({\varvec{q}}+{\varvec{k}},t\right).$$

Finally, the spatial Fourier transform of Eq. (2) leads to:

10 $$\frac{\partial {F}_{s}({\varvec{q}},t)}{\partial t}-i\left({\varvec{q}}-{\varvec{k}}\right)\cdot {{\varvec{v}}}_{0}{\text{exp}}\left(-i\omega t\right){F}_{s}\left({\varvec{q}}+{\varvec{k}},t\right)=-D{q}^{2}{F}_{s}\left({\varvec{q}},t\right).$$

In the case of a uniform flow with the velocity of ${{\varvec{v}}}_{0}$ instead of ultrasonic vibration (i.e., $k=\omega =0$), Eq. (10) reduces to the same equation derived by Berne and Pecora^20^ as:

11 $$\frac{\partial {F}_{s}({\varvec{q}},t)}{\partial t}-i{\varvec{q}}\cdot {{\varvec{v}}}_{0}{F}_{s}\left({\varvec{q}},t\right)=-D{q}^{2}{F}_{s}\left({\varvec{q}},t\right).$$

With the initial condition of ${F}_{s}\left({\varvec{q}},0\right)=1$, we have ${F}_{s}\left({\varvec{q}},t\right)={\text{exp}}(-D{q}^{2}t){\text{exp}}(i{\varvec{q}}\cdot {{\varvec{v}}}_{0}t)$.

At sufficiently low US frequencies (i.e., less than 1 GHz) in water, as the medium, $q\gg k$. Therefore, the Eq. (10) can be simplified as:

12 $$\frac{\partial {F}_{s}({\varvec{q}},t)}{\partial t}-i{\varvec{q}}\cdot {{\varvec{v}}}_{0}{\text{exp}}\left(-i\omega t\right){F}_{s}\left({\varvec{q}},t\right)=-D{q}^{2}{F}_{s}\left({\varvec{q}},t\right).$$

By solving the Eq. (12) with the initial condition of ${F}_{s}\left({\varvec{q}},0\right)=1$,${F}_{s}$ can be obtained as:

13 $${F}_{s}\left({\varvec{q}},t\right)={\text{exp}}\left\{-D{q}^{2}t\right\}{\text{exp}}\{-\frac{{\varvec{q}}\cdot {{\varvec{v}}}_{0}}{\omega }{\text{exp}}(-i\omega t)\}.$$

Assume that ${{\varvec{v}}}_{0}={{\varvec{r}}}_{0}\omega$, where ${r}_{0}$ is the amplitude of vibration, Eq. (13) can be rewritten as:

14 $${F}_{s}\left({\varvec{q}},t\right)={\text{exp}}\left\{-D{q}^{2}t\right\}{\text{exp}}\{-{\varvec{q}}\cdot {{\varvec{r}}}_{0}{\text{exp}}\left(-i\omega t\right)\}.$$

From Berne and Pecora^20^, the homodyne correlation function can be obtained as:

15 $${F}_{2}\left({\varvec{q}},t\right)={\langle N\rangle }^{2}[1+\left|{F}_{s}{\left({\varvec{q}},t\right)}^{2}\right|]+\langle \delta N\left(0\right)\delta N\left(t\right)\rangle = {\langle N\rangle }^{2}[1+Re({\text{exp}}\left\{-D{q}^{2}t\right\}{\text{exp}}\left\{-2{\varvec{q}}\cdot {{\varvec{r}}}_{0}{\text{exp}}\left(-i\omega t\right)\right\}]+\langle \delta N\left(0\right)\delta N(t)\rangle$$

Therefore, in the homodyne experiment, the autocorrelation function for the lag time, $\tau$, can be written as:

16 $$g_{2} \left( \tau \right) = A\left[ {1 + B{\text{exp}}\{ - 2Dq^{2} \tau \} {\text{exp}}\{ - 2qr_{0}^{\prime } {\text{cos}}\left( {\omega \tau } \right)\} } \right],$$

where $r_{0}^{\prime } = r_{0} {\text{cos}}\alpha$, and $\alpha$ is the angle between the ${r}_{0}$ and $q$. The B is called intercept, related to light-collection efficiency, and can be omitted by normalizing the autocorrelation function^24^. Therefore, the normalized autocorrelation function (NACF) can be written as:

17 $$NACF=\frac{{g}_{2}\left(\tau \right)-A}{B}={\text{exp}}\left\{-2D{q}^{2}\tau \right\}{\text{exp}}\left\{-2q{r\mathrm{^{\prime}}}_{0}{\text{cos}}\left(\omega \tau \right)\right\}.$$

## Experimental

**Figure 1:**
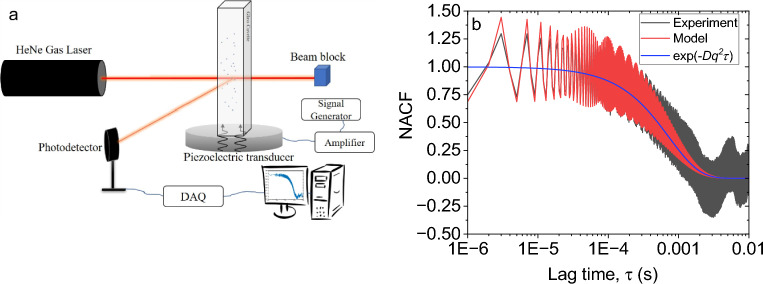
(**a**) Schematic of the experimental apparatus comprising a DLS setup and US system connected to the sample via a piezoelectric transducer and glass cuvette. (**b**) Normalized autocorrelation function (NACF) for 200 nm silica particles subjected to 255 kHz and 300 mV US.

The experimental setup, as schematically shown in Fig. 1a, comprises a HeNe gas laser (HNL150L, Thorlabs GmbH, Germany, $\lambda =633 {\text{nm}}$) with a power of 30 W, a photodetector (APD130A2/M, Thorlabs GmbH, Germany) detecting the scattered light at an angle of 90°, data acquisition system (BNC-2110, National Instruments, USA, sampling rate $=$ 1 MS/s) connected to a computer. Piezoelectric transducers (STEMINC-PIEZO, Davenport, IA, USA) with different resonance frequencies (40 kHz, 255kHz, 780 kHz, 2.34 MHz, and 5.4 MHz) were used and attached to the glass cuvette (inside dimensions of 10 × 10 × 40 mm^3^) using a two-component latex glue (UHU Endfast Plus300, Bühl, Germany). The RF signal was generated using a function generator (SDG1062X, SIGLENT, Shenzhen, China) and was amplified by an RF amplifier (VBA100-30, Vectawave, UK). We used silica nanospheres (nanoComposix, CA, USA) in different diameters (80, 200, 500, and 1000 nm) as colloidal dispersions diluted in milli-Q water. PNIPAM microgel particles with 5 mol% cross-linker content (BIS) were synthesized using precipitation polymerization. For detailed information see our previous works^25,26^. All samples were prepared in concentrations between 0.005 and 0.01 wt%. A commercially available DLS system (LS Instrument, Switzerland) was used to measure the hydrodynamic diameter of PNIPAM microgels due to temperature changes. The generated ultrasonic waves are in continuous form. The DLS measurements (for 10–20 s) were started with the start of the US and also after 2–3 min from the starting time, in the case of compact particles. In the case of microgels, since their size change over time was the subject of the study, the DLS measurements (10 s) started immediately after the starting of the US and were done every minute until 5 min. The experiments with the US were performed at 22 °C. Temperature rise in dispersions due to imposing the US was negligible in the case of compact particles since the actuation took place less than 3 min and in the case of microgels was less than 2 °C after 5 min of actuation which is far below the VPTT. Temperature measurements were done by thermo-couple (PT-100) with an uncertainty of ± 0.5 °C.

## Results and discussion

**Figure 2:**
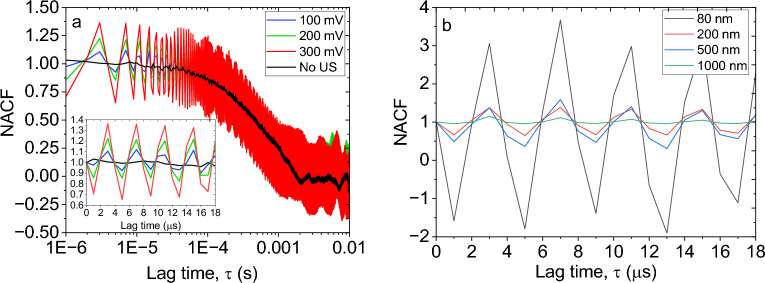
Normalized autocorrelation function (NACF) of (**a**) 200 nm particles at different input voltages and (**b**) particles with different diameters at 300 mV input voltage. In both cases, the frequency of US is set to 255 kHz.

According to Eq. (17), the photon intensity correlation function exhibits an oscillatory behavior. Such behavior previously was observed for particles in alternating electric fields at very low frequencies^4^. From the modified NACF, the diffusion coefficient due to Brownian motion and hence the particle size can be acquired independently of the US effect. Moreover, the oscillatory component in Eq. (17) specifies both the frequency and amplitude of the particle's vibration. According to Fig. 1a, the angle between the light detection and transducer's vibration is 90°. However, the complex acoustic field inside the closed cuvette comprises a series of standing compression/expansion waves that make the particles vibration both horizontally and vertically. The possibility of leaking waves from the walls of the cuvette makes it more complicated to determine $\alpha$ experimentally. Therefore, it needs more investigation and at this stage, we only report the $r_{0}^{\prime }$. Using a MATLAB script, the experimental results are fitted by the diffusion term in linear cumulant analysis together with the oscillatory term in Eq. (17). All error bars were calculated using the standard deviation of ten measurements in each data point. The NACF in both the experiment and model (shown in Fig. 1b) for 200 nm silica particles subjected to 255 kHz US shows a good agreement. However, the oscillations damp gradually in the model while the experiments show a fixed amplitude of oscillations. As the lag time increases, the autocorrelation function starts to include information about slower dynamics. This can include longer-range diffusion, aggregate formation, and other processes that occur on longer timescales. These slower processes may lead to more pronounced oscillations in the autocorrelation function, especially if particles are undergoing more complex and correlated motions. One example in our case might be acoustic streaming which has a time scale of milliseconds to seconds^27^. Thus, the growing NACF due to the aforementioned reasons at larger lag times may be superimposed by the NACF from Eq. (17) leading to the relatively constant amplitude of oscillation. Nevertheless, NACF at 255 kHz gives us valuable information about the frequency and amplitude of vibrations (Fig. 2). The frequency of these oscillations– which is shown clearer using the linear time scale in the graph inset– is equal to the input frequency of the US (Fig. 2a). In order to exclude the possible coupling of the US with the electrical components of the detection system we used pure water as the sample. The resulting NACF in Figure S1 shows no specific frequency of oscillation matching with the input frequency (i.e., 255kHz). Moreover, in order to possibly couple the US with the laser, at least the wavelength of both should be in the same range (~ 600 nm). In order to have an acoustic wavelength in this range (inside the water), the wave frequency would have to be in the range of 2 GHz. This is 3 orders of magnitude higher than the current studied frequencies. According to Fig. 2a, the curve without US presents a mean value around which the data recorded in the presence of US fluctuates. The amplitude of oscillations increases by increasing the input voltage although the frequency and phase remain constant. Figure 2b shows how the particles with different sizes behave under the same US properties. Particles of smaller sizes, such as those with 80 nm diameter, demonstrate significantly greater oscillation amplitude compared to their larger counterparts, like those with a size of 1000 nm. However, despite the amplitude difference, both small and large particles exhibit the same frequency and phase of oscillation. The extracted amplitude of vibration based on our model in Eq. (17) and Fig. 2b is shown in Table 1 for all particles. Despite that the order of magnitude of the amplitudes in Table 1 seems reasonable, unfortunately, we have no other experimental method to confirm the values.

**Table 1:** Vibration amplitude of nanoparticles subjected to 255 kHz and 300 mV US extracted from NACF.

Particle diameter (nm)	80	200	500	1000
Vibration ${{{r}}\boldsymbol{^{\prime}}}_{0}$ (nm)	61.95 ± 8.35	10.7 ± 1.6	14.06 ± 2.01	4.68 ± 0.66

**Figure 3:**
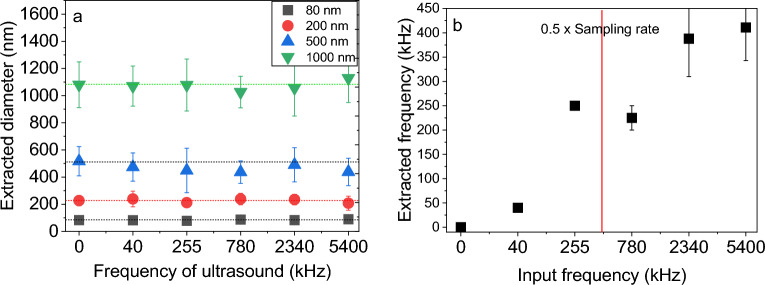
Extracted (**a**) diameter of silica particles and (**b**) frequency of particle vibration from NACF.

Based on our theory and experimental results, we can assert that the estimation of particle size can be accurately determined regardless of the frequency and amplitude of the US used (Fig. 3a). An example of NACF for 200 nm particles subjected to different frequencies is shown in Figure S2 of the supporting information. The frequency of US, which is identical to that of the particles, can be extracted from NACF provided that the data acquisition system has enough sampling rate. Figure 3b shows that at frequencies higher than half of the sampling rate (which is 500 kHz) the oscillations cannot be captured in the correct frequency complying with the Nyquist–Shannon sampling theorem^28^. Now, the question is how acoustic streaming does not interfere with the DLS results. Generally, when a liquid is subjected to US, a flow field develops due to the absorption of ultrasonic waves by the liquid viscosity. This flow may influence the diffusion of particles by increasing the translational velocity of particles leading to a wrong size estimation. However, in our experiments, the diffusion time scales for the range of particle sizes investigated are considerably shorter than the time scales associated with acoustic streaming. As a result, acoustic streaming does not influence the extracted diffusion coefficient. From the work by Leung et al.^11^ one can realize that when there is a uniform flow having a low velocity (less than 1 cm/s), the particle size can be obtained with an acceptable error using the conventional intensity correlation functions. Acoustic streaming often has a velocity range of less than 1 mm/s^29^. That is why for the sub-micrometer particles, the diffusion is fast enough that the effect of acoustic streaming is negligible. Furthermore, particles that are subjected to ultrasound waves in a closed system (i.e., the acoustic field comprises complex standing waves with several nodes) experience acoustic radiation force and viscous Stokes drag force. These two forces compete with each other. When the particle is large, acoustic radiation force is dominant and leads to straight movement of the particle toward a node. On the other hand, the small particle, dominated by drag force from acoustic streaming rolls, moves in circular paths^30^. All of the aforementioned scenarios are related to large time scales ($\ge {\text{ms}}$)^27^. So far, it has not been possible to resolve the µs-ranged particle motion experimentally. Our results in Fig. 2 show that while particles are moving, they experience vibration in excitation frequency. Larger (i.e., heavier) particles vibrate with lower amplitude (Table 1).

**Figure 4:**
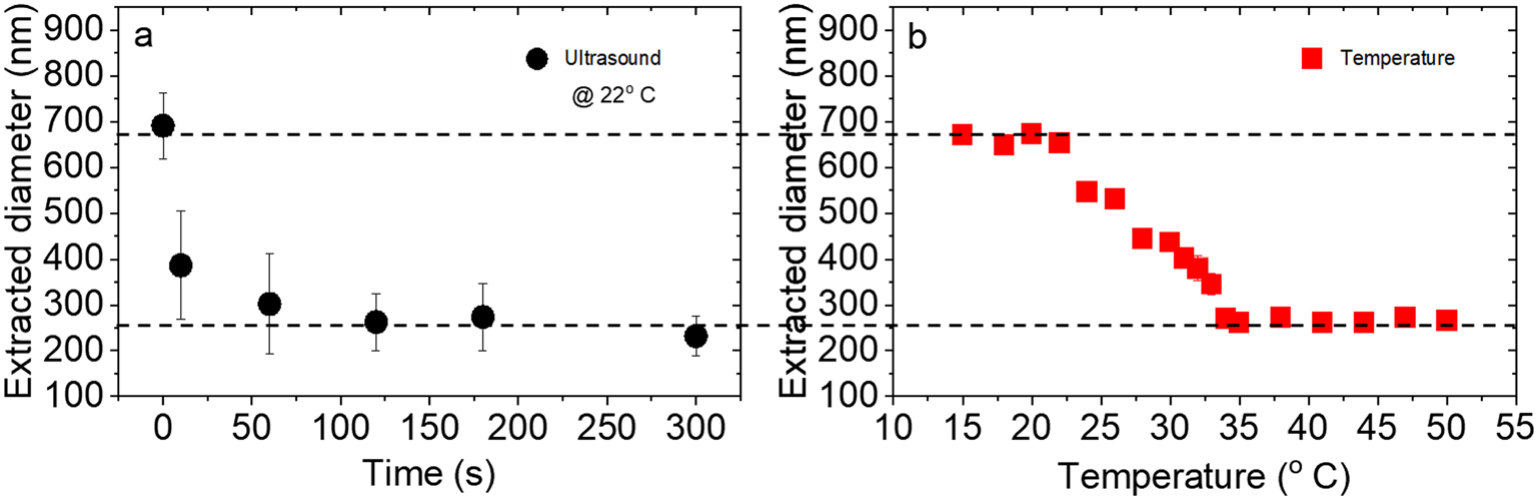
Extracted diameter of PNIPAM microgel (5 mol% cross-linker content), exhibiting the VPT due to both (**a**) ultrasonic actuation at room temperature (22 °C) and (**b**) temperature change. The US frequency and input voltage were set to 5.4 MHz and 400 mV respectively.

Taking advantage of the analyzed reference system, we studied the US-induced VPT of PNIPAM microgels. As shown in Fig. 4a, the extracted hydrodynamic diameter of microgels (from 690 nm at swollen state) decreases (to 232 nm at collapsed state) due to imposing US over time. This is the same size as achieved with increasing temperature above the VPTT (Fig. 4b) but with much faster kinetics. The problem with the temperature, as a stimulus, is that in reality, it takes time until the liquid dispersion in the cuvette reaches a uniform value reasonable enough for a measurement. There are works^31–33^ that tried to investigate the kinetics of PNIPAM response to fast temperature change using a high heating rate, leading to VPT in less than a minute. Wrede et al. showed in the case of using a pressure jump microgels are able to collapse in 10 ms^34^. When we use ultrasound as the stimulus, the waves transfer through the whole medium with the speed of sound. The reduction of microgel size after 10 s of actuation implies fast dehydration upon imposing the US. However, the larger error bars during the transient size change may arise due to two factors: the inhomogeneity of the particle sizes (collapsed and swollen microgels) within the measured spot and the measurement duration (about 10 s), which falls within the continuous actuation period. Because the extracted diameters were averaged through the 10 s of measurement.

In the case of compact particles, we assume that they vibrate with a given amplitude and frequency. However, when the particles are soft (e.g., microgels), their vibration amplitude and frequency may differ from those of compact particles due to dissipation. As shown in Figure S3, the amplitude of oscillation of microgels at the collapsed state (i.e., 300 s after stimulation) has been decreased compared with the swollen state (i.e., at starting time of stimulation). This effect may arise due to the stiffness of microgels in these two states which requires a systematic investigation. Since our data acquisition system cannot resolve the 5 MHz frequency, we cannot comment much on that. However, it seems in both cases oscillations are in the same frequency. The hydrodynamic diameter of microgels was extracted from the mean value of the NACF at a respective time.

## Conclusion

In this work, we developed a DLS characterization of silica particles under the influence of the US. In the theory part, we rewrite the continuity equation based on the time-dependent velocity of particles due to ultrasonic waves. Then, we derive a modified intensity autocorrelation function for dilute nano-spheres undergoing Brownian diffusion as well as ultrasonic vibration. The resulting model gives valuable information about the particle vibrational behavior in addition to the Brownian diffusion coefficient. The experimental work is performed using a US-DLS setup for silica particles of different sizes (from 80 nm to 1 µm) at different US frequencies (40 kHz to 5.4 MHz) and amplitudes. The findings with silica particles indicate that any potential disturbances, such as acoustic streaming, which could impact the size estimation of particles in DLS experiments, can be excluded. Therefore, the particle size can be correctly estimated even with the conventional fitting parameters for the mean value of oscillating autocorrelation data points. Furthermore, it is possible to extract the particle vibration amplitude and frequency in addition to their size successfully. This is because the frequency of particle vibration is high enough that the vibration and diffusion time scales are decoupled. This method is important for the characterization of macromolecules and polymers whose size and behavior subjected to US are of interest. We used the developed setup to evaluate the shrinking behavior of PNIPAM microgels subjected to US. Our findings demonstrate that PNIPAM microgels are a notable example of acousto-responsive polymer networks. Their VPT in response to the US holds great potential for their application in drug delivery systems. In addition, the US is a much faster trigger than temperature change. Our previous work^17^ demonstrated that higher ultrasound frequencies and amplitudes accelerate turbidity evolution in linear PNIPAM due to increased energy absorption. The same principle may apply to PNIPAM microgels, exploring their response to different ultrasound frequencies and amplitudes. Our broader aim is to leverage DLS to characterize particle systems influenced by ultrasound, with microgels serving as a confirmatory example. Apart from the case of acousto-responsive microgels, the concept of tracking vibrating particles through DLS opens the door to the development of an innovative method for micro-rheology. Depending on the stiffness of the particles, the oscillation of ACF may differ and it may be possible to obtain the elastic modulus of those particles according to the amplitude and frequency of vibration.

### Supplementary Information

Supplementary Figures.

## Data Availability

The data used and/or analyzed during the current study is available from the corresponding author on reasonable request.
