# Supplementary material for: Dynamic light scattering for particle characterization subjected to ultrasound: a study on compact particles and acousto-responsive microgels
Source: Sci Rep. 2024 Jan 10;14:989. doi: 10.1038/s41598-024-51404-0 (PMC10781767; doi:10.1038/s41598-024-51404-0)
Supplement: Supplementary file 1 — Supplementary Figures. [file 41598_2024_51404_MOESM1_ESM.docx]

**Supporting Information:**

**Dynamic Light Scattering for Particle Characterization Subjected to Ultrasound: A Study on Compact Particles and Acousto-responsive Microgels**

Sebastian Stock, Regine von Klitzing, and Amin Rahimzadeh^*^

Soft Matter at Interfaces, Institute for Condensed Matter Physics, Technische Universität Darmstadt, Hochschulstraße 8, 64289 Darmstadt, Germany

^*^ Email address: [Amin.rahimzadeh@pkm.tu-darmstadt.de](mailto:Amin.rahimzadeh@pkm.tu-darmstadt.de)

Figure S1. NACF for pure water subjected to 255 kHz and 300 mV ultrasound and without US.

Figure S2. NACF of 200 nm Si particles subjected to the US in three different frequencies.

Figure S3. NACF of PNIPAM microgels cross-linked by 5 mol% BIS subjected to 5.4 MHz US immediately after starting and 300 s after starting actuation.
